# An update of a systematic review and meta‐analyses exploring flavours in intervention studies of e‐cigarettes for smoking cessation

**DOI:** 10.1111/add.16736

**Published:** 2024-12-19

**Authors:** Nicola Lindson, Jonathan Livingstone‐Banks, Ailsa R. Butler, David T. Levy, Phoebe Barnett, Annika Theodoulou, Caitlin Notley, Nancy A. Rigotti, Yixian Chen, Jamie Hartmann‐Boyce

**Affiliations:** ^1^ Nuffield Department of Primary Care Health Sciences University of Oxford Oxford UK; ^2^ Cancer Prevention and Control Program Georgetown University‐Lombardi Comprehensive Cancer Center Washington DC USA; ^3^ Centre for Outcomes Research and Effectiveness, Research department of Clinical, Educational and Health psychology University College London UK; ^4^ Addiction Research Group, Norwich Medical School University of East Anglia UK; ^5^ Tobacco Research and Treatment Center, Department of Medicine, Massachusetts General Hospital Harvard Medical School Boston MA USA; ^6^ Department of Integrative Oncology British Columbia Cancer Research Institute Vancouver British Columbia Canada; ^7^ Department of Health Promotion and Policy University of Massachusetts Amherst Amherst MA USA

**Keywords:** e‐cigarettes, flavours, nicotine, smoking cessation, systematic review, tobacco

## Abstract

**Aims:**

To determine patterns of e‐cigarette flavour use (sweet, tobacco, menthol/mint) in interventional studies of e‐cigarettes for stopping smoking, and to estimate associations between flavours and smoking/vaping outcomes.

**Methods:**

Update of secondary data analyses, including meta‐analyses subgrouped by flavour provision and narrative syntheses, incorporating data from January 2004 to February 2024. Eligible studies were identified from a Cochrane review. Studies provided adults who smoked cigarettes with nicotine‐containing e‐cigarettes for smoking cessation and provided data on e‐cigarette e‐liquid flavour use. Outcomes included participants' flavour use measured at any time, plus smoking abstinence, abstinence from all tobacco or commercial nicotine products and allocated product use at 6 months or longer, reported as risk ratios with 95% confidence intervals. We assessed risk of bias using the Cochrane Risk of Bias 1 tool.

**Results:**

We included 25 studies (*n* = 16 748); 21 contributed to subgroup meta‐analyses and 18 provided flavour choices. We judged 15 studies at high, seven at low and three at unclear risk of bias. In studies where participants had a choice of flavours, some switching between flavours occurred (five studies). A preference for sweet (including fruit) flavours over tobacco and menthol was indicated (in 6 of 11 studies); however, there were differences across studies. Subgroup meta‐analyses showed no clear associations between e‐liquid flavours provided and smoking cessation or study product use. One included study randomised participants to two different flavour conditions and found similar cessation rates and long‐term e‐cigarette use between arms at 12 months.

**Conclusions:**

Some people using e‐cigarettes to quit smoking switch between e‐cigarette flavours during a quit attempt. Sweet flavours may be preferred overall, but this may differ depending on context. Based on intervention studies, there is no clear association between the use of e‐cigarette flavours and smoking cessation or longer‐term e‐cigarette use, possibly due to a paucity of data.

## INTRODUCTION

The 2024 update of the Cochrane living systematic review of e‐cigarettes (EC) for smoking cessation found that EC can help people stop smoking combustible cigarettes [1]. Nicotine e‐liquids are available in many flavours. However, evidence on whether and how flavours influence the effectiveness of EC is limited [2]. Although EC are less harmful than combustible cigarettes they are not risk‐free, and there have been debates around flavour restrictions as a way to minimise youth vaping [3]. Additionally, concerns persist regarding the impact of regulating flavours on the appeal, use and effectiveness of EC as a stop‐smoking tool [3].

Observational data from the United States (US) has found fruit to be the most popular e‐liquid flavours and that people are more likely to successfully transition away from smoking if they use non‐tobacco flavoured EC [4, 5]. However, this data needs to be triangulated in RCTs.

In 2022, we published secondary syntheses of intervention studies included in the Cochrane living review discussed above [6]. We incorporated 16 studies identified up to January 2022. None directly compared different flavours between randomised groups and there was no clear evidence that e‐liquid flavours affected smoking or vaping at 6 months or longer. There was also no clear evidence of the popularity of particular e‐liquid flavours over others. The limited nature of the data synthesised hampered our ability to draw clear conclusions. We set out to update these analyses to incorporate the most recent data and to continue to inform flavour policy discussions.

## METHODS

This article updates an earlier version of this review [6]. We present a summary of the key methods and findings, with a particular focus on content added since the last version. For a more detailed version of this review see Data S1. We pre‐specified our methods: https://osf.io/HPBYW/.

### Objectives

Our goal was to (1) investigate patterns of EC flavour use (sweet, tobacco, menthol/mint) where participants were provided with a choice of flavours; (2) investigate whether the effectiveness of using nicotine EC to stop smoking was associated with flavour of EC used; and (3) investigate whether the long‐term (6 months or longer) use of the allocated study product was associated with flavour of nicotine EC used.

### Searches, screening and data extraction

We identified eligible studies from our Cochrane review (i.e. intervention studies in which people who smoked combustible cigarettes were provided with or asked to use EC, published from 2004) up to February 2024 [1]. To be included here, studies had to report information on e‐liquid flavour use and report at least one of the following outcomes at 6 months or longer: (1) combustible cigarette abstinence; and (2) use of study product (EC or comparator intervention). Eligibility for the Cochrane review was determined in duplicate, whereas a single reviewer determined eligibility for this review.

We used relevant data previously extracted in duplicate for the Cochrane review along with new data gathered for this review [1], including an additional outcome: participants abstinent from all tobacco or commercial nicotine products (excluding medicinal nicotine replacement therapy and including EC) at 6 months follow‐up or longer. Additional data was extracted by one reviewer and verified by another. We categorised e‐liquid flavours as: tobacco only; menthol/mint only; sweet only (including fruit, candy and dessert flavours); unflavoured only; choice of tobacco or menthol/mint; choice of tobacco, menthol/mint or sweet.

We applied risk of bias judgements from the Cochrane review, which were conducted in duplicate using the Cochrane risk of bias 1 tool [1]. Studies were deemed low, unclear or high risk of bias overall.

### Syntheses

When studies offered participants a range of flavours, we extracted this information and reported it narratively. To investigate associations between flavours and our outcomes of interest, we subgrouped our existing Cochrane meta‐analyses by the e‐liquid flavour(s) provided in the studies. We conducted analyses using RevMan 5.4, using I^2^ to assess subgroup differences. An I^2^ greater than 50% was deemed substantial and potentially indicative of an association between flavours and the outcome of interest. Pooled subgroup effects were calculated using random effects analyses and presented as risk ratios (RR) with 95% CI. We extracted results of any within‐study analyses by flavours allocated/chosen in the studies and synthesised them descriptively.

## RESULTS

Of the 90 studies identified in our Cochrane review to February 2024, 25 were eligible for this review; nine of these new to this review (see Table 1 and Data S1). Of the 25, 21 were RCTs included in subgrouped meta‐analyses, and 18 reported providing participants with a choice of flavours (some studies qualified for both categories). Fifteen studies were judged to be at high risk of bias, three at unclear risk and seven at low risk. One study randomised participants to different e‐liquid flavours (tobacco only e‐liquids versus choice of sweet, menthol/mint or tobacco flavour e‐liquids) and reported on smoking cessation and long‐term product use.

**TABLE 1:** Characteristics of included studies.

Study ID	Device type	Total *n* baseline	Flavours provided	Intervention investigated	Study design	Length of follow‐up (months)	Overall risk of bias judgement	Country	Population characteristics
Auer et al.^a^ [7]	Refillable	1246	Choice of sweet, tobacco or menthol	C (EC vs. counselling)	RCT	6	High	Switzerland	People who smoke combustible cigarettes
Begh 2021	Refillable	325	Choice of sweet, tobacco or menthol	C (EC vs. standard care)	RCT	8	High	UK	People who smoke combustible cigarettes with no plans to stop
Bullen 2013	Cig‐a‐like	657	Tobacco only	C (EC vs. nicotine patches vs. placebo EC)	RCT	6	Low	New Zealand	People who smoke combustible cigarettes and willing to quit
Caponnetto 2013	Cig‐a‐like	300	Tobacco only	C (EC vs. lower nicotine EC vs. non‐nicotine EC)	RCT	12	Unclear	Italy	People who smoke combustible cigarettes
Caponnetto 2023^a^	Refillable	220	Choice of tobacco or menthol	C (EC vs. heated tobacco)	RCT	6	High	Italy	People who smoke combustible cigarettes
Carpenter 2023^a^	Pod	638	Choice of sweet, tobacco or menthol	C (EC vs. no intervention)	RCT	6	High	USA	People who smoke combustible cigarettes
Cobb 2021	Cartridge	520	Choice of tobacco or menthol	C (EC nicotine 2 strengths; non‐nicotine EC; cigarette substitute)	RCT	6	Low	USA	People who smoke combustible cigarettes
Dawkins 2020	Refillable	80	Choice of sweet, tobacco or menthol	C (EC vs. UC)	Prospective cohort	6	High	UK	People who smoke combustible cigarettes. Recruitment at homeless centres
Eisenberg 2020	Cig‐a‐like	376	Tobacco only	C (EC + counselling vs. non‐nicotine EC + counselling vs. counselling only)	RCT	6	Low	Canada	People who smoke combustible cigarettes and motivated to quit
Ely 2013	Cig‐a‐like	48	Choice of sweet, tobacco or menthol	S (All used EC)	Prospective cohort	6	High	USA	People who want to quit combustible cigarettes or switch to EC
Hajek 2019^a^	Refillable	886	Tobacco only	C (EC vs. NRT)	RCT	12	Low	UK	People who smoke combustible cigarettes
Hajek 2022	Refillable	1140	Choice of sweet or tobacco	C (EC vs. NRT)	RCT	6	Low	UK	People who smoke combustible cigarettes, 12–24 weeks pregnant
Halpern 2018	Cig‐a‐like	6006	Choice of sweet, tobacco or menthol	C (UC + EC; UC + EC + NRT + bupropion or varenicline; UC + EC + NRT + bupropion or varenicline + incentives; as before plus financial incentive)	RCT	12	High	USA	People who smoke and employees and their spouses that used Vitality wellness programs
Holliday et al. [8]	Refillable	80	Choice of sweet, tobacco, mint/menthol, or unflavoured	C (EC vs. no intervention)	RCT	6	High	UK	People who smoke combustible cigarettes with periodontitis
Klonizakis 2022^a^	Cartridge	248	Choice of tobacco or menthol	C (EC vs. non‐nicotine EC; EC vs. NRT)	RCT	6	Unclear	UK	People who smoke combustible cigarettes
Lee 2018	Cig‐a‐like	30	Tobacco only	C (EC vs. nicotine patches)	RCT	6	Low	USA	People who smoke and presented to the anaesthesia preoperative clinic for elective surgery 3 or more days before surgery
Lucchiari 2020	Cig‐a‐like	210	Tobacco only	C (nicotine EC vs. non‐nicotine EC)	RCT	12 but data only available at 6	High	Italy	Participants are 55 years or more and have smoked at least 10 combustible cigarettes a day for the past 10 years
Myers Smith 2022	Refillable	135	Choice of sweet, tobacco or menthol	C (EC vs. NRT)	RCT	6	Low	UK	People who smoke combustible cigarettes and find quitting difficult
Polosa 2015	Refillable	71	Choice of sweet, tobacco or menthol	S (All used EC)	Prospective cohort	12	High	Italy	People who smoke combustible cigarettes, making first purchase at vape shop
Pope 2024^a^	Pod	972	Choice of sweet, tobacco or menthol	C (EC vs. UC)	RCT	6	High	UK	People attending the emergency department who smoked tobacco daily
Pratt 2022^a^	Cartridge	240	Choice of tobacco or menthol	C (EC vs. no intervention)	RCT	6	High	USA	People who smoke combustible cigarettes with serious mental illness
Price 2022^a^	Refillable	871	Choice of sweet, tobacco or menthol	S (All offered EC)	Single‐arm intervention study	12	High	UK	People who smoke combustible cigarettes from lower socioeconomic groups
Pulvers et al. [9]	Pod	186	Choice of sweet, tobacco or menthol	C (EC versus no intervention)	RCT	6	High	USA	African American and Latinx people who smoke combustible cigarettes
Russell 2021	Pod	426	Choice of sweet, tobacco or menthol	C (NRT; EC with nicotine salt e‐liquid pods; EC with freebase nicotine e‐liquid pods)	RCT	6	Unclear	UK	People who smoke combustible cigarettes
Xu 2023^a^	Pod	837	(1) Tobacco only; (2) Choice of sweet, tobacco or menthol	C (Tobacco flavour EC vs. choice of flavour EC vs. quit advice)	RCT	12	High	USA	People who smoke combustible cigarettes

Refer to Data S1 for included study references. Abbreviations: C, comparison; EC, electronic cigarettes; NRT, nicotine replacement therapy; UC, usual care; UK, United Kingdom; USA, United States of America; S, single arm.

^a^ New to this update.

### Studies offering a choice of flavours

Of the 18 studies offering a choice of flavours, four provided participants with a choice of tobacco or menthol/mint flavours, one with a choice of sweet or tobacco flavours and the remaining studies provided a choice of sweet, menthol/mint or tobacco flavours. Eleven provided a breakdown of flavours used. Six (United States‐ and United Kingdom‐based) studies showed sweet flavours were more popular than menthol/mint and tobacco flavours (Begh 2021, Dawkins 2020, Hajek 2019, Myers Smith 2022, Price 2022, Xu 2023) (Table 1). One Swiss study found 75% of participants preferred sweet and menthol/mint flavours (no further breakdown) over tobacco [7]. Two studies based in Italy showed over 80% of participants preferred tobacco flavour versus other flavours offered [10, 11]. One UK study found no clear preference between sweet, tobacco, and mint/menthol flavours [8], whereas a US study that recruited Latinx and African‐American participants reported 54% chose menthol/mint, 17.6% tobacco and 28% sweet flavours [9]. Xu 2023 found that participants preferred sweet flavours overall, but that menthol/mint e‐liquid was most popular among those who usually smoked menthol cigarettes, whereas tobacco and sweet flavours were the most popular among those who usually smoked non‐menthol cigarettes [12].

**FIGURE 1:**
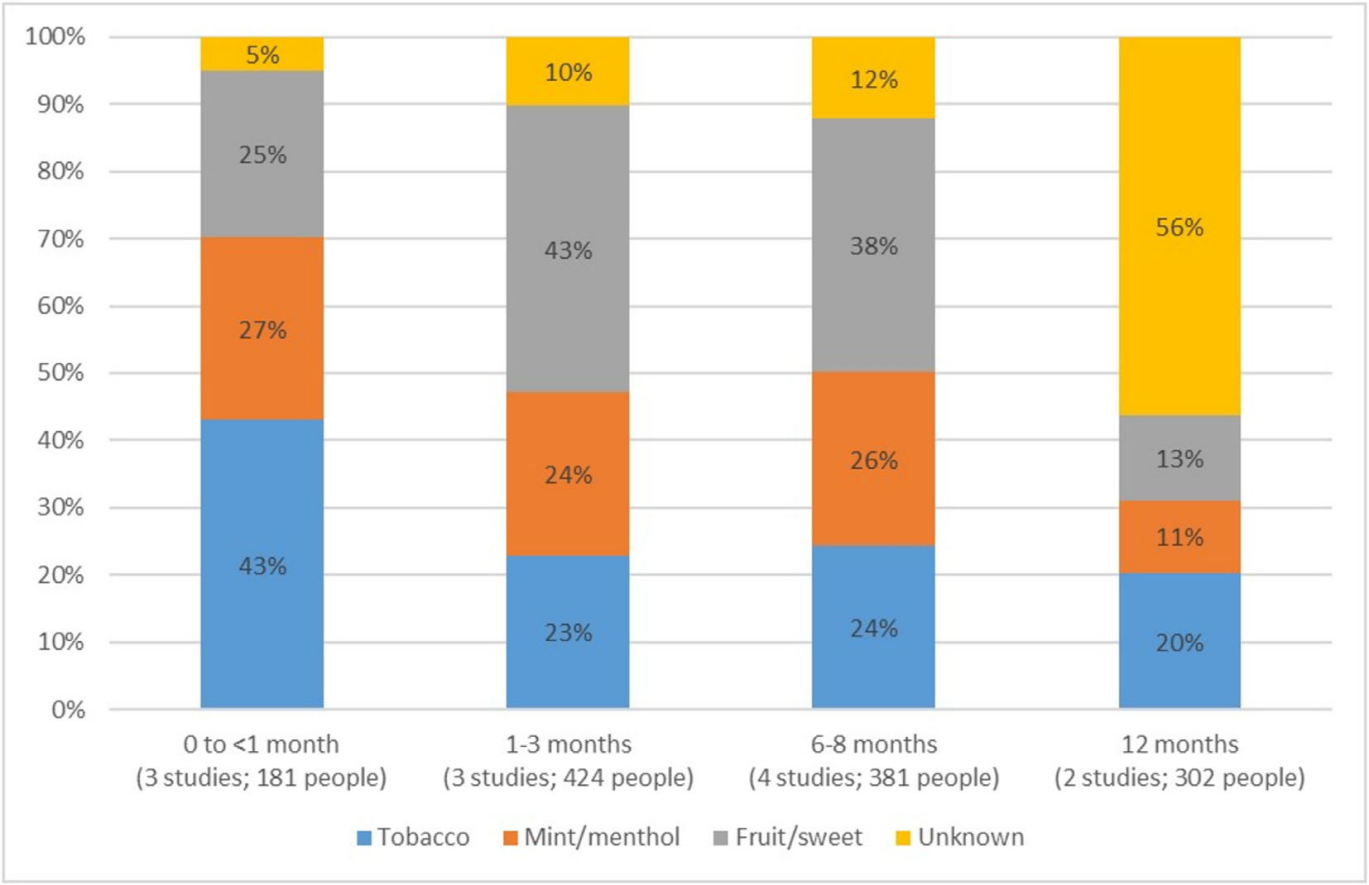
Flavour choice over time in five studies providing options including tobacco, mint/menthol and sweet flavours.

Figure 1 (new to this update) summarises flavour use over time in five studies that provided flavour use information at multiple timepoints and offered a choice of tobacco, menthol/mint and sweet EC flavours. This data is provided for descriptive purposes only (it has not been meta‐analysed) and cannot account for differences in the characteristics of included studies or participants. Although tobacco flavour was most used at the earliest time point, this appeared to decline substantially at later time points, whereas use of menthol remained fairly stable between zero and 6/8 months, and use of sweet flavours increased slightly in the same period.

**FIGURE 2:**
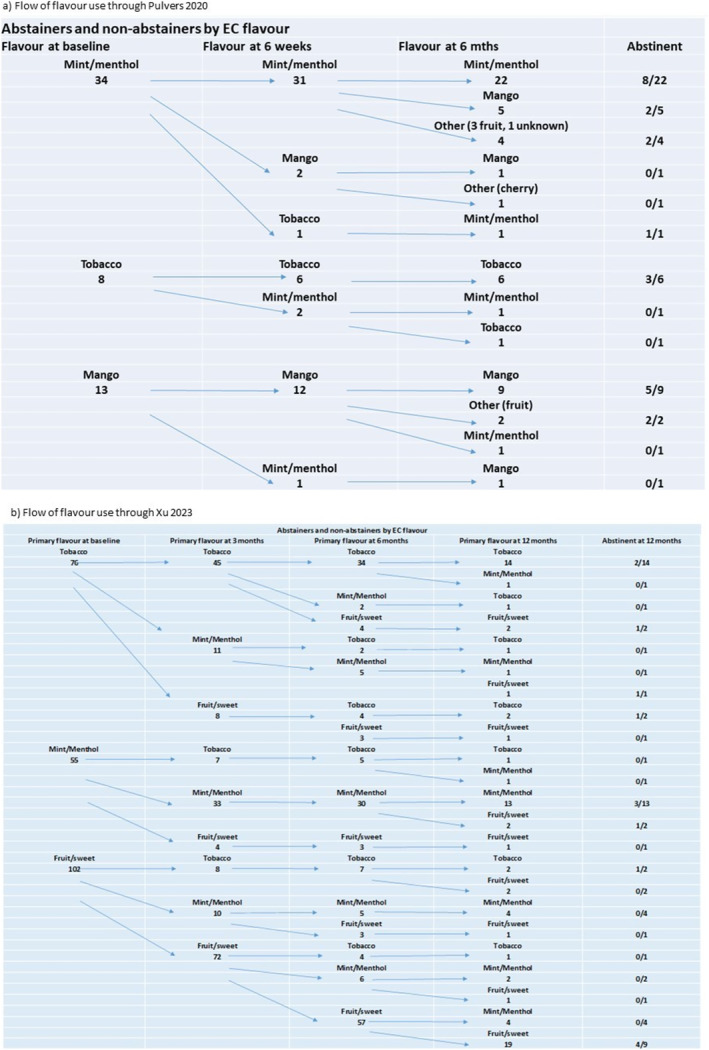
EC flavour use over time among participants in (a) Pulvers *et al*. [9], (b) Xu 2023. [12]. Arrows illustrate the flow of flavour choice and switching behaviour (only including participants that provided data at follow‐up points). (a) At baseline and 6‐week follow‐up participants were provided with mango, mint, menthol or tobacco flavours. At 6‐month follow‐up participants were self‐sourcing flavours and so additional flavours were being used, as specified; (b) up to 6 months participants were supplied with tobacco, mint, menthol, mango, creme, fruit or cucumber by the study; at 12 months participants were self‐sourcing EC supplies.

Using individual participant data from two studies, we mapped flavour switching behaviour (Figure 2) [9, 12]. In both studies, the majority of people stayed with their initial flavour choice throughout; however, a substantial minority switched between flavours over time. Pulvers *et al*. [9] (included in previous update) found a slight decrease in menthol use and an increase in fruit flavours, with stable tobacco flavour use. Xu 2023 (new to this update) found a slight increase in menthol/mint use, whereas the use of sweet and tobacco flavours stayed consistent [12].

### Associations between flavours and outcomes

One study, new to this update and conducted by an EC company, randomised participants into three groups. Two groups were relevant when comparing flavours—tobacco flavoured e‐liquid versus choice of sweet, tobacco or menthol e‐liquids [12]. The study reported similar 12‐month, 30‐day point prevalence quit rates in the tobacco (51/285; 17.9%) and flavour choice (40/281; 14.2%) groups. At 12‐month follow‐up, 83 of 261 (31.8%) participants in the tobacco group had used an EC in the last 30 days versus 91 of 261 (34.9%) in the flavour choice group.

**FIGURE 3:**
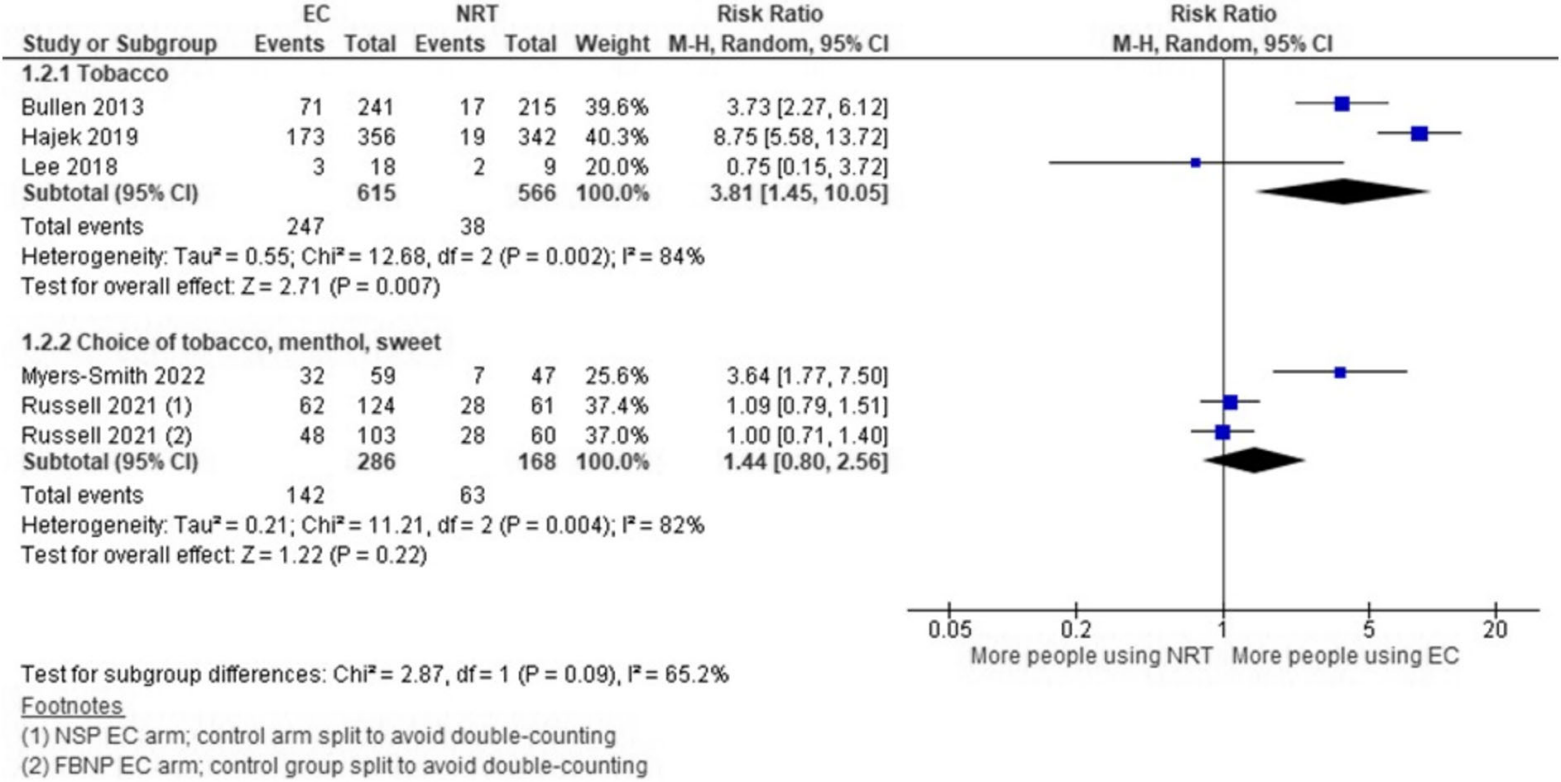
Study product use at 6 months or longer, EC versus NRT*.* EC, electronic cigarettes; FBNP, free base nicotine pods; NSP, nicotine salt pods; NRT, nicotine replacement therapy.

No clear evidence emerged from our subgrouped meta‐analyses (21 studies across analyses) that flavour moderated smoking abstinence or long‐term product use. The I^2^ for subgroup differences suggested substantial statistical heterogeneity (65.2%) for the comparison ‘nicotine EC versus nicotine replacement therapy (NRT)’ and long‐term study product use outcome (5 studies) (Figure 3). However, substantial within subgroup statistical heterogeneity was also detected and all subgroup analyses were limited by imprecision. Only one study measured abstinence from both combustible cigarettes and EC across study arms [13]. This was a small study with small numbers of participants quitting. Of the two participants abstinent at 8 months in the EC intervention arm both were using sweet flavours (as opposed to menthol or tobacco flavours) early in the study. No participants quit in the standard care arm.

Five studies that provided participants with a choice of flavours supplied data on the flavour choices of participants who quit smoking versus those who did not achieve abstinence. No definitive patterns of flavour use emerged.

## DISCUSSION

This update builds on a previous systematic review investigating the role of e‐liquid flavours in people who smoked [6]. It extends the ongoing Cochrane living review of EC for smoking cessation [1].

The previous version of the review was largely inconclusive, revealing no specific e‐liquid flavour preferences across studies nor a clear association between EC flavours and smoking abstinence or longer‐term EC use [6]. However, there was limited evidence of some people switching e‐liquid flavours during a quit attempt. Overall, there was substantial uncertainty in our findings. This update incorporates nine additional studies, bringing the total to 25. One was a new RCT directly comparing two flavour conditions [12]. There was no clear evidence to suggest that particular flavours were associated with better quit rates or greater long‐term use of EC. At this update most studies providing information on flavour use provided evidence that sweet flavours were preferred; however, there were some notable exceptions. Two Italian studies found tobacco flavour e‐liquids were preferred over other flavours [10, 11], and two studies found evidence that people who had a previous history of smoking mentholated combustible cigarettes were more likely to prefer menthol flavoured e‐liquids [9, 12]. One of the studies was carried out in Latinx and African‐American participants who more commonly smoke menthol cigarettes than the remaining US population [14]. Therefore, there may be cultural differences in e‐liquid flavour use that need to be considered. In addition, there is now increased evidence that although some people continue using the same e‐liquid flavour throughout a quit attempt, there are also numerous people who switch between flavours, with the most common switching being away from tobacco to other flavours.

The primary outcome of this review was abstinence from combustible cigarettes, but several other outcomes, including abstinence from both combustible cigarettes and EC, warrant reporting in future studies. For this update, we investigated this as an additional outcome, as quitting all products is the ultimate aim to achieve the maximum health benefit. However, only one of our included studies measured this across all participants in the study [13]. Because of the small sample size and limited cessation rate in this study, the findings for this outcome were inconclusive. Future studies investigating EC in people who smoke should also collect detailed information on flavour use over time and investigate any potential moderating effects on study outcomes. Ideally more RCTs should be carried out randomising participants to differing flavour conditions because this is the only study design that can be used to establish causality. This is vital as regulation of EC flavours should take into account both the role of flavours in achieving smoking abstinence and the uptake of EC in young people who have not previously smoked, to balance risks and benefits.

Our approach is based on established systematic review methods, with thorough search, study selection and data collection methods [1]. For reasons of pragmatism screening for this review was carried out by a single reviewer. However, as initial eligibility was determined in duplicate for the Cochrane review and eligibility for this review relied on the reporting of outcomes extracted in duplicate for the Cochrane review, this is deemed to be a minimal risk. There are uncertainties in our conclusions because of the limited data available. Although other studies have also indicated preferences for sweet flavoured e‐liquids over other flavours [4, 15], further research could change conclusions, underscoring the need for continuous monitoring of emerging data.

## AUTHOR CONTRIBUTIONS

**Nicola Lindson:** Conceptualization (equal); data curation (equal); formal analysis (lead); funding acquisition (equal); investigation (equal); methodology (equal); project administration (lead); resources (equal); software (equal); supervision (lead); validation (lead); visualization (equal); writing—original draft (lead). **Jonathan Livingstone‐Banks:** Conceptualization (supporting); data curation (equal); formal analysis (supporting); funding acquisition (equal); investigation (supporting); methodology (supporting); project administration (supporting); supervision (supporting); validation (supporting); visualization (equal). **Ailsa R. Butler:** Conceptualization (equal); data curation (supporting); formal analysis (supporting); investigation (supporting); methodology (supporting); project administration (supporting); writing—original draft (supporting). **David T. Levy:** Conceptualization (supporting); funding acquisition (equal); methodology (supporting). **Phoebe Barnett:** Data curation (supporting). **Annika Theodoulou:** Data curation (supporting). **Caitlin Notley:** Data curation (supporting). **Nancy A. Rigotti:** Conceptualization (supporting). **Yixian Chen:** Data curation (supporting). **Jamie Hartmann‐Boyce:** Conceptualization (equal); funding acquisition (equal); methodology (equal); supervision (supporting); writing—original draft (supporting).

## DECLARATIONS OF INTEREST

N.L., A.R.B., D.T.L., P.B., A.T. and YC have nothing to declare. C.N. has received an honorarium from Vox Media for filming a ‘nicotine explainer’ on the role of nicotine in addiction. C.N. was co‐PI on the CoSTED trial (Pope 2024; NIHR129438). J.H.B. has received research consultancy funding from the Food and Drug Administration and the Truth Initiative. J.L.B. is an author of a paper reporting a trial included in this review (Pope 2024). N.A.R. has received royalties from UpToDate, for chapters on electronic cigarettes. Outside the topic of EC, she has consulted for and received research grants from Achieve Life Sciences.

## Supporting information


**Data S1.** Supplementary Information.

## Data Availability

Data sharing not applicable to this article as no new datasets were generated.
