# Supplementary material for: An update of a systematic review and meta‐analyses exploring flavours in intervention studies of e‐cigarettes for smoking cessation
Source: Addiction. 2024 Dec 19;120(4):770–8. doi: 10.1111/add.16736 (PMC11907327; doi:10.1111/add.16736)
Supplement: Supplementary file 1 — Data S1. Supplementary Information. [file ADD-120-770-s001.docx]

Supplemental file 1: Full review update 2024

An exploration of flavours in intervention studies of e-cigarettes for smoking cessation: secondary analyses of a systematic review with meta-analyses

Nicola Lindson, PhD^a^

Jonathan Livingstone-Banks, PhD^a^

Ailsa R Butler, DPhil^a^

David T. Levy, PhD^b^

Phoebe Barnett, PhD^c^

Annika Theodoulou, MClinSc^a^

Caitlin Notley PhD^d^

Nancy A Rigotti, MD^e^

Yixian Chen, PhD^f^

Jamie Hartmann-Boyce, DPhil^a,g^

a Nuffield Department of Primary Care Health Sciences, University of Oxford, Oxford, UK. Email: [ailsa.butler@phc.ox.ac.uk](mailto:ailsa.butler@phc.ox.ac.uk); [nicola.lindson@phc.ox.ac.uk](mailto:nicola.lindson@phc.ox.ac.uk); [annika.theodoulou@phc.ox.ac.uk](mailto:annika.theodoulou@phc.ox.ac.uk); [jonathan.livingstone-banks@phc.ox.ac.uk](mailto:jonathan.livingstone-banks@phc.ox.ac.uk)

b Georgetown University-Lombardi Comprehensive Cancer Center, Cancer Prevention and Control Program, Washington, DC, USA. Email: [dl777@georgetown.edu](mailto:dl777@georgetown.edu)

c Centre for Outcomes Research and Effectiveness, Research department of Clinical, Educational and Health psychology, University College London. Email: [Phoebe.barnett@ucl.ac.uk](mailto:Phoebe.barnett@ucl.ac.uk)

d Addiction Research Group, Norwich Medical School, University of East Anglia. c.notley@uea.ac.uk

e Tobacco Research and Treatment Center, Department of Medicine, Massachusetts General Hospital, Harvard Medical School, Boston, Massachusetts, USA. Email: [NRIGOTTI@PARTNERS.ORG](mailto:NRIGOTTI@PARTNERS.ORG)

f Department of Integrative Oncology, British Columbia Cancer Research Institute, Vancouver, British Columbia, Canada. Email: [ycchen@bccrc.ca](mailto:ycchen@bccrc.ca)

g Department of Health Promotion and Policy, University of Massachusetts Amherst, Amherst, MA, USA. Email: jhartmannboy@umass.edu

Acknowledgements

We would like to thank our funders (see funding section) and study investigators who provided data contributing to this manuscript: Professor Penny Cook, Aimee Hodgkinson, Dr Nicholas Goldenson, Dr Sarah Pratt, Dr Francesca Pesola, Dr Katie Myers Smith, Professor Kim Pulvers, Dr Paul A Bateman, Dr Rachna Begh, Dr Pasquale Caponnetto and Professor Riccardo Polosa.

Declarations of competing interest

NL, ARB, DTL, PB, AT, YC: None

CN has received an honorarium from Vox Media for filming a 'nicotine explainer' on the role of nicotine in addiction. CN was co-PI on CoSTED trial (Pope 2024; NIHR129438).

JHB has received research consultancy funding from the Food and Drug Administration and the Truth Initiative.

JLB is an author of a paper reporting a trial included in this review (Pope 2024).

NAR has received royalties from UpToDate, Inc., for chapters on electronic cigarettes. Outside the topic of e-cigarettes, she has consulted for and received research grants from Achieve Life Sciences.

Funding

Research reported in this publication update was supported by the National Cancer Institute of the National Institutes of Health (NIH) and FDA Center for Tobacco Products (CTP) under Award Number 2U54CA229974. The content is solely the responsibility of the authors and does not necessarily represent the official views of the NIH or the Food and Drug Administration. Support for the parent review comes from Cancer Research UK (PRCPJT-Nov22/100012). The previous update was also funded by an Oxford University Public Policy Challenge Grant and infrastructure funding for the Cochrane Tobacco Addiction Group provided by the National Institute for Health and Care Research (NIHR). The views and opinions expressed therein are those of the authors and do not necessarily reflect those of the Systematic Reviews Programme, NIHR, National Health Service (NHS) or the Department of Health.

**ABSTRACT**

**Aims**

To investigate patterns of e-cigarette flavour use (sweet, tobacco, menthol/mint) in interventional studies of e-cigarettes for stopping smoking, and to estimate associations between flavours and smoking/vaping outcomes.

**Methods**

Update of secondary data analyses, including meta-analyses subgrouped by flavour provision and narrative syntheses, incorporating data from January 2024 to February 2024. Eligible studies were identified from a Cochrane review. Studies provided adults who smoked cigarettes with nicotine-containing e-cigarettes for smoking cessation and provided data on e-cigarette e-liquid flavour use. Outcomes included participants’ flavour use measured at any time, plus smoking abstinence, abstinence from all tobacco or commercial nicotine products, and allocated product use at 6 months or longer, reported as risk ratios with 95% confidence intervals. We assessed risk of bias using the Cochrane Risk of Bias 1 tool.

**Results**

We included 25 studies (n=16,748); 21 contributed to subgroup meta-analyses and 18 provided flavour choices. We judged 15 studies at high, seven at low, and three at unclear risk of bias. In studies where participants had a choice of flavours, some switching between flavours occurred (5 studies). A preference for sweet (including fruit) flavours over tobacco and menthol was indicated (in 6 of 11 studies); however, there were differences across studies. Subgroup meta-analyses showed no clear associations between e-liquid flavours provided and smoking cessation or study product use. One included study randomised participants to two different flavour conditions and found similar cessation rates and long-term e-cigarette use between arms at 12 months.

**Conclusions**

Some people using e-cigarettes to quit smoking switch between e-cigarette flavours during a quit attempt. Sweet flavours may be preferred overall, but this may differ depending on context. Based on intervention studies there is no clear association between the use of e-cigarette flavours and smoking cessation or longer-term e-cigarette use, possibly due to a paucity of data.

**BACKGROUND**

E-cigarettes (EC) are a popular aid for quitting smoking. The most recent update of our Cochrane living systematic review of ‘Electronic cigarettes for smoking cessation’ shows high certainty evidence that more people successfully quit smoking using nicotine-containing EC than using nicotine replacement therapy (NRT).(1) EC consumables (e.g., e-liquids) are available in a variety of flavours that can be matched to either a person’s favoured cigarette type (i.e., tobacco or menthol) or something completely different, such as fruits, candies or desserts. There are ongoing policy debates about restricting EC flavour options, particularly as a mechanism to prevent youth vaping. While population surveys have attempted to examine whether EC flavours affect smoking cessation,(2-6) there is very little evidence available on how EC flavours influence quitting in clinical trials.

There are theoretical reasons that flavour might impact the effects of ECs on quitting smoking. Using a flavour that matches a user’s flavour of combustible cigarettes could boost the likelihood of successful quitting if people are less likely to miss their combustible cigarettes and are thus less likely to relapse. It is also possible that using an EC flavour different from a person’s usual cigarettes could increase the novelty and desirability of the product, and be less likely to reinforce the established addiction, thereby reducing cigarette dependence. A systematic review by Gades et al. explored differences between EC flavours and found evidence that people who smoked combustible cigarettes and used non-tobacco flavoured e-liquids were more likely to have reduced or quit smoking than those using tobacco or unflavoured e-liquids.(7) EC users valued the ability to switch between flavours and it was one of the main reasons given for EC use; following health and smoking cessation.(7) In addition, population surveys have found that using non-tobacco flavoured EC has been associated with a greater likelihood of stopping combustible cigarette use.(2-6) A UK cross-sectional survey found that people typically started out using tobacco-flavoured EC and transferred to sweet or food-flavoured products.(8)

On the other hand, a systematic review conducted by Liber et al. 2023, examining the association between EC flavours used and smoking cessation found the evidence to be inconclusive.(9) As did a recent experimental study that randomised 84 people who smoked to either EC with fruit/sweet flavoured e-liquids or EC with unflavoured e-liquids for one week and assessed cigarette cravings at one week follow-up.(10)

Any potential benefits of a range of flavour options for smoking cessation must be balanced against concerns that the availability of flavours, such as fruits, candies, and desserts, make EC more desirable to people who have never smoked, especially young people, and will result in more people using EC recreationally as opposed to as a quitting aid. A systematic review by Notley et al. provided some evidence that people under the age of 18 years enjoyed flavoured EC products and had a preference for fruit and other sweet flavours.(11) Concerns about young people’s use of flavours have led to bans, or considerations of bans, on the sale of particular EC flavours in some jurisdictions.(12) In people who have smoked combustible cigarettes, another important consideration is that the use of flavoured, as opposed to unflavoured EC products, may increase the length of time EC are used after a smoking quit attempt. This could have positive implications if longer-term EC use reduces the risk of relapse to smoking combustible cigarettes but could be a cause for concern if longer-term use leads to harm.

In 2022, we published the first version of this review, concluding that in line with the evidence highlighted above, some people using EC to quit smoking do appear to switch between EC flavours.(13) There did not appear to be any clear association between EC flavours and smoking cessation or longer-term EC use, possibly due to a paucity of data. Therefore, we concluded that findings may change as more evidence becomes available.

To inform ongoing discussions around flavour policies, and incorporate all available recent evidence, we have updated the 2022 systematic review.(13) We investigated whether EC flavours were associated with tobacco smoking quit success or longer-term use of EC in adults, when provided as stop-smoking aids in intervention studies of EC for smoking cessation that met the eligibility criteria for our Cochrane review.(1) Our objectives were as follows:

1. To investigate patterns of EC flavour use (sweet, tobacco, menthol/mint) where participants were provided with a choice of flavours;
2. To investigate whether the effectiveness of using nicotine EC to stop smoking was associated with flavour of EC used;
3. To investigate whether the long-term (6 months or longer) use of study product was associated with flavour of nicotine EC used.

**METHODS**

**Searches, screening, and data extraction**

This synthesis extends our living systematic review of EC for smoking cessation. More detailed information on search methods, eligibility criteria, and data extraction is available in that review (and summarised in supplementary file 2).(1) The methods for the analyses reported in this paper were pre-registered on Open Science Framework (<https://osf.io/HPBYW/>). We included randomized controlled trials (RCTs) or randomized cross‐over trials, in which people who smoked combustible cigarettes were randomized to EC or any control condition. Additionally, we included uncontrolled intervention studies where all participants received an EC intervention, though these studies were excluded from our meta-analyses. Specific to this review, we only included studies identified in searches between January 2004 and February 2024, which provided information on EC flavour use and reported on at least one of the following outcomes:

- Long-term cessation of combustible cigarettes (at 6 months follow-up or longer; we also refer to this as ‘abstinence’ in the text which pertains specifically to abstinence from combustible cigarettes)
- Proportion of people still using study product (EC or comparator intervention) at longest follow‐up (at 6 months follow-up or longer).

Although we carry out screening in duplicate for our main review, a single reviewer carried out eligibility screening for the syntheses reported in this paper. Data were extracted in duplicate as part of the parent review. A single reviewer revisited the studies eligible for this review to extract relevant flavour information and data pertaining to an additional outcome for this update - the number of participants who stopped using all tobacco or commercial nicotine products (excluding medicinal NRT, and including e-cigarettes) at 6 month follow-up or longer. This was then checked by a second reviewer. We categorised flavours into subgroups: tobacco only; menthol/mint only; sweet only (including fruit, candy and dessert flavours); unflavoured only; choice of tobacco or menthol/mint; choice of tobacco, menthol/mint or sweet.

Risk of bias judgements followed the criteria specified in the parent review.(1, 14) Studies were categorized as low risk of bias overall if all domains were deemed low risk, high risk of bias if any domain was deemed high risk, or unclear risk where no domains were deemed high risk but at least one was deemed unclear risk.

**Syntheses**

To fulfil objective 1, where studies offered participants a range of flavours, a single reviewer extracted any information reported on participants’ flavour choices.

To investigate associations between flavours and our outcomes of interest (objectives 2 and 3), we subgrouped existing meta-analyses from our Cochrane review by the flavours of EC provided in the included studies, for the following primary comparisons:

- Nicotine EC vs NRT
- Nicotine EC vs non-nicotine EC
- Nicotine EC vs behavioural support only or no intervention

We updated our existing analyses using Cochrane’s RevMan 5.4 software where there were sufficient studies and data. We investigated differences between subgroups, using I^2^ for subgroup differences, when more than one study and subgroup were included in an analysis. . An I^2^ greater than 50% was deemed substantial and potentially indicative of an association between flavours and the outcome of interest. We also examined the pooled estimates for each subgroup and assessed whether their interpretation differed across groups. We calculated pooled subgroup effect estimates as risk ratios (RR) with 95% confidence intervals (CI). In the parent review,(1) analyses were carried out using fixed effects models; however, in the next update analyses will be updated to random effects to reflect clinical and methodological variance across studies included in meta-analyses. Thus, for this paper, we present random effects analyses, with fixed effect analyses available in supplementary file 2. Had there been sufficient data we would have meta-analysed data for our new outcome - the number of participants who stopped using all tobacco or commercial nicotine products (excluding medicinal NRT, and including e-cigarettes), subgrouping by flavours provided. However, only one included study provided this data for relevant study arms.(15)

We also extracted the results of any analyses authors had carried out of our outcomes (smoking cessation, study product use and cessation of all tobacco and commercial nicotine products; all at 6 months or longer) by flavours allocated/chosen. We synthesised this data without meta-analysis, i.e., narratively and using descriptive figures.

**RESULTS**

**Included studies**

Through February 2024, our literature searches identified 90 studies eligible for inclusion in the parent review.(1) Twenty-five of these met our criteria for inclusion in this review (see supplemental file 2; figure S1 for flow diagram); nine that were new to this update (see Table 1). Of the 25, 21 RCTs were eligible for inclusion in relevant meta-analyses, and 18 studies reported providing participants with a choice of EC flavour (some studies were included in both types of synthesis). From the latter, we attempted to extract further information on participant choice and the impact of flavour on our outcomes of interest. Table 1 includes summary information on all included studies. Fifteen of the eligible studies were judged to be at high risk of bias overall, three at unclear risk and seven at low risk (further information on reasons for judgements is available in supplemental file 2 (Table S1) and the parent review).(1) We identified one study that randomised participants to different e-liquid flavours and reported on two of our pre-specified outcomes (cessation and long-term product use).(16) Participants were randomised to EC with tobacco e-liquids, EC with a choice of sweet, tobacco or menthol flavoured e-liquids or advice to quit smoking only.

**Studies offering a choice of flavours (objective 1)**

Table 2 reports the information we extracted on the flavours offered by the 18 studies that provided participants with a choice of flavours. Four of the studies provided participants with a choice of tobacco or menthol/mint flavours, one with a choice of sweet or tobacco flavours and the remaining studies provided a choice of sweet, menthol/mint or tobacco flavours.

Eleven of the 18 studies offering a choice of flavours provided a breakdown of flavours used at one or more time points. Six of these studies (conducted in the US and UK) showed a higher popularity of sweet over menthol/mint and tobacco flavours,(15-20) with another study, conducted in Switzerland, finding that 75% of participants used a flavour other than tobacco (sweet or menthol/mint, with no preference breakdown across these categories).(21) The only two studies conducted in Italy both found a preference for tobacco flavour – just over 80% of participants chose tobacco flavour in both (one study offered a choice of tobacco and menthol and the other a choice of tobacco, menthol and sweet).(22, 23) One study (UK-based) observed no clear demarcation in preferences between sweet, tobacco and mint/menthol flavours.(24) The final study reporting on preferences reported that most participants opted for menthol/mint (54%) over tobacco (17.6%) and sweet flavours (28%).(25) Further to this, Xu 2023 investigated flavour choice moderated by whether participants smoked mentholated or non-mentholated cigarettes on entry into their study.(16) When looking at the study arm where participants were given a choice of flavours (where there was a greater preference for sweet flavours overall), menthol/mint flavours were most popular among participants who usually smoked mentholated cigarettes (52%, compared to 9% choosing tobacco flavours and 39% choosing sweet flavours), whereas tobacco and sweet flavours were similarly popular among participants who usually smoked non-mentholated cigarettes (43% and 46% respectively, compared to 11% menthol/mint flavours).(15, 24)

Figure 1 provides a descriptive summary of the use of flavours over time in five studies that provided flavour use information at more than one time point and offered a choice of tobacco, menthol/mint and sweet EC flavours.(15, 16, 18, 23, 25) This data is provided for descriptive purposes only (it has not been meta-analysed) and cannot account for differences in the characteristics of included studies or participants. Due to the substantial missing data at the 12-month time point it is hard to interpret trends; however, there is some indication of a decline in the use of tobacco flavours from one month follow-up onwards (declining from 43% at the zero to < one month time point to between 24% and 20% at later time points). The proportion of participants using menthol/mint flavours appeared to remain fairly stable between zero and six/eight months (between 27% and 24%), and the proportion of participants using sweet flavours appeared to increase in the same period (from 25% to 38%).

Using individual participant data supplied by two study teams, we were able to map the flavour switching behaviour of participants in those studies (see Figure 2). Pulvers 2020 (who recruited African American and Latinx participants in the US) found that a substantial minority of participants switched the flavour of EC they used during the study.(25) The proportion using menthol flavour decreased slightly over time, while the proportion using mango or other fruit flavours increased and the proportion using tobacco remained stable; absolute numbers were small for all scenarios. Similarly, Xu 2023 found that, although most people stayed with their initial flavour choice throughout, notable switching occurred between flavours in a smaller subset.(16) The proportions of participants using tobacco and fruit flavours stayed relatively stable across the study; however, the use of menthol/mint increased slightly.

**Associations between flavours and outcomes of interest (objectives 2 and 3)**

One study, new to this update, randomised participants to different flavour choices; this is the only analysis able to assess the possibility of a causal relationship between flavours and outcomes.(16) This study randomised participants to three groups, two of which are relevant when comparing randomised flavour conditions - EC with tobacco flavour pods and EC with a choice of sweet, tobacco or menthol pods. At 12 month follow-up, 51/285 (17.9%) participants in the tobacco pod study arm and 40/281 (14.2%) participants in the flavour choice arm reported 30-day point prevalence smoking abstinence (RR 1.26; 95% CI 0.86 to 1.84). Also at 12 month follow-up (six months after study EC provision had ceased), 83/261 (31.8%) of the tobacco flavour study arm had used an EC in the last 30 days and 91/261 (34.9%) of the flavour choice study arm (RR 0.91; CI 0.72 to 1.16). The investigators highlighted that not all participants randomised to the tobacco flavour arm solely used tobacco flavour EC and some participants purchased their own pods in other flavours. However, use of tobacco flavour as the primary flavour was high in the tobacco arm across the one, three and six month follow-ups; over 75% of participants used a tobacco flavour at each of these time points, compared to approximately 32% using tobacco flavours at each of these time points in the choice of flavours arm.(16)

For the majority of subgrouped meta-analyses (including 21 studies across analyses), subgrouping by our specified flavour categories did not show evidence of effect moderation (see Table 3 for summaries of feasible subgroup analyses). The only analysis where there was an I^2^ suggesting substantial statistical heterogeneity between groups (I^2^ = 65.2%), was for the comparison ‘Nicotine EC versus NRT’ and the outcome of long-term study product use (5 studies; Figure 3). Three studies were included in a ‘tobacco’ flavour subgroup,(27-29) and two studies in a ‘choice of tobacco, menthol/mint or sweet’ group.(18, 30) The ‘tobacco’ subgroup provided evidence that more participants were using EC at long-term follow-up than were using NRT; whereas the ‘choice’ subgroup had a substantially smaller point estimate favouring higher long-term EC use, with CIs also encompassing the potential for higher long-term NRT use as well as no difference in product use between study groups. However, this finding should be treated with caution as there was substantial statistical heterogeneity within subgroups (I^2^ = 84% for ‘tobacco’ subgroup; I^2^ = 82% for ‘choice of tobacco, menthol/mint, sweet’ subgroup), and other differences between studies could have been driving apparent differences in effects. All of our subgroup analyses were limited by imprecision due to small numbers of events within analyses and subgroups. Future eligible studies could change interpretation of subgroup differences. See supplementary file 2 for additional forest plots (figures S2.1-S4.1), as well as the results of the subgrouping of fixed effects analyses (figures S5.1-S7.1).

We contacted the author teams of the 11 studies that offered a choice of flavours to see if they could provide further information on potential moderating effects of flavours chosen. Five provided some additional information related to flavour use and smoking abstinence.(15, 16, 18, 23, 25) The flavour most used by cigarette abstainers varied between studies (see Figure 4). In one UK study, the majority of people who were abstinent from smoking were using sweet flavours, with smaller, very similar numbers using menthol/mint and tobacco flavours.(18) In a US study, the participants who chose sweet (mango) flavour at baseline were most likely to be abstinent from cigarettes at six-month follow-up (54% quit versus 38% menthol/mint and 37% tobacco flavours; see Figure 3a); most abstainers were using sweet/fruit, or menthol/mint flavours at follow-up (Figure 4).(25) A second US study reported that 30-day point prevalence quit rates at 12-month follow-up were similar across primary flavours chosen in the relevant study arm (using complete case analysis): tobacco 4/25 (16%); menthol/mint 5/28 (18%); sweet 7/36 (19%),(16) and a UK study, Begh 2021, found that of the four participants who quit, two were using sweet flavours, one tobacco flavour and one menthol/mint flavour.(15) In an Italian study, sweet flavours appeared to be used the least by cigarette abstainers at follow-up, with tobacco the most popular flavour.(23) Three of the studies also provided the number of people using each EC flavour who were not abstinent ≥ six month follow-up (Figure 4).(15, 16, 25) Among the non-abstinent group, flavour use was evenly matched in one study, with nine participants using tobacco; 10 using mint; and nine using sweet flavours.(15) In the others, tobacco flavour seemed to be less popular at follow-up, with mint/menthol the most popular in one,(25) and sweet the most popular in the other.(16) As in our subgroup analyses, it is important to treat the data on abstinence and flavours with caution as the groupings of participants and the events occurring within them were relatively small for all studies, and are observational in nature.

Only Begh 2021 reported data on our third, new outcome; cessation of all tobacco and commercial nicotine products. This study provided a choice of sweet, menthol and tobacco flavours and found that 1.2% (2/164) of the EC study arm and 0% (0/161) of the standard care arm were abstinent from tobacco and EC at 8 month follow-up.(15) Both of the participants abstinent from cigarettes and e-cigarettes were using sweet flavours earlier in the study. This was a relatively small study (N=325), with participants unmotivated to quit smoking, and so quit rates were low. Though some additional studies reported this outcome for the intervention arm only, none reported data on this outcome broken down by flavours used.

**DISCUSSION**

This paper reports updated findings from syntheses conducted as an extension to our Cochrane living systematic review of EC for smoking cessation.(1, 13) We explored the flavour use of people using EC to help them to quit smoking and investigated any moderating effect of EC flavour on the success of EC as a smoking cessation aid, the likelihood of quitting the use of both combustible cigarettes and EC, and the likelihood of using EC long-term. Most studies that reported on participants’ flavour preferences indicated that sweet EC liquids (including fruit and dessert flavours) were the most popular. However, there were some variations across studies; e.g., studies in Italy showed some indication of a preference for tobacco flavours and participants who were smoking mentholated cigarettes at baseline appeared to show a preference for menthol/mint flavours. This study was carried out in the US and recruited Latinx and African‐American participants, who more commonly smoke menthol cigarettes than the remaining US population.(26) In all studies that considered flavour use over time, there appeared to be flavour switching; however, in most cases, it was hard to distinguish the full extent due to a lack of individual participant data. In the two US studies where it was possible to track individual use, there was notable experimentation with different flavours in some participants, although others used the same flavour throughout.(16, 25)

Only one study meeting our eligibility criteria randomised participants to different flavour conditions: participants receiving tobacco flavour EC pods versus participants having a choice of tobacco, menthol/mint, or sweet flavour EC pods.(16) There was no evidence of a clear difference in smoking abstinence rates between groups at the 12 month follow-up.(16) Subgrouping analyses from the parent review by the flavour of EC offered did not provide evidence that cessation or long-term product use were associated with the flavours provided. However, these findings are based on small numbers of studies and participants, and subject to confounding, thus are likely to change as more evidence becomes available. Only one study reported the outcome of both smoking and EC cessation and results were inconclusive.(15) A subset of studies provided data on the flavours used by participants who were ultimately abstinent from tobacco at long-term follow-up; yet no consistent flavour pattern emerged across them.(15, 18, 23, 25) These findings should again be treated with caution for the aforementioned reasons.

Our approach is based on data from a high-quality, established living systematic review.(1) The searches and processes used to identify studies are thorough and involve searching for unpublished, as well as published literature, aiming to minimise bias and maximise our chances of identifying all relevant literature. For pragmatic reasons, screening and data extraction for this sub-study were conducted by a single author, potentially increasing the opportunity for human error. However, this manuscript has been reviewed by all authors, the majority of whom are experts in the field and many who are also authors of the parent review; the latter of whom know the included studies well. For these secondary analyses we made the decision to group EC liquid flavours into sweet (incorporating fruit), menthol/mint and tobacco subgroupings. This was based on the current regulatory climate, i.e. the groupings most regularly discussed when considering and implementing flavour bans, and the limited data currently available (splitting sweet flavours into fruit and other sweet flavours, for example, would have limited the data in each category and reduced statistical power). However, we recognise the breadth of the flavour market and will consider whether these groupings are still appropriate for any future updates.

All syntheses included in this paper are based on small numbers of studies and participants. Few intervention studies have provided information on EC flavour use and preference. Therefore, our investigation and conclusions are severely limited by the lack of available primary data, particularly the lack of individual participant data. In addition, the single subgrouped meta-analysis that suggested a potential association between long-term EC use and flavour exhibited considerable statistical heterogeneity within subgroups, making it difficult to draw meaningful conclusions. At the time of writing, only one published RCT (conducted and funded by an EC company) has investigated our outcomes of interest and directly compared different EC flavour conditions.(16) This RCT compared offering a choice of flavours versus offering one flavour, rather than directly comparing particular flavours. Consequently, most of the evidence reported in this review relates to associations rather than causal relationships. A final important limitation is that these data derive from studies where participants were recruited and consented to use EC to aid in quitting smoking. Consequently, these studies may not accurately assess whether uptake of EC as a quit aid is associated with flavour availability.

Other literature on EC flavours presents disparate findings. Data from a large US longitudinal cohort study (PATH), collected between 2014 and 2016, found that the most popular flavours of EC were fruit flavours, in line with the findings of this review.(31) A subsequent study using the same dataset between 2014 and 2018 showed that people who smoked were more likely to transition from smoking to EC use if they used non-tobacco flavoured EC.(6) Data collected on US purchase transactions between 2013 and 2017 revealed that adult cigarette smokers tended to purchase tobacco flavour EC or e-liquids more than others.(32) EC consumers appeared to be loyal to their preferred flavour. A New Zealand study of 32 participants, who completed at least four interviews, provided participants with an EC starter kit but required them to source e-liquids of their choosing.(33) The majority initially selected a tobacco flavoured e-liquid, with the remainder choosing fruit, menthol/mint, dessert/sweet and non-alcoholic beverage flavours in approximately equal proportions. Most participants were using the same flavour at study exit; however, many had experimented with different flavours at the beginning of the study. In contrast, a study carried out in Australia in February 2022, found that fruit flavours were the most used e-liquids among all e-cigarette users, regardless of their smoking status or age.(34) Finally, a systematic review of any study that investigated differences in EC flavours published up to August 2020, found evidence that flavour preferences had changed over time,(7) with a preference for the more traditional cigarette flavours of tobacco and menthol shifting toward sweet flavours. This appeared to be true even in people using combustible cigarettes along with EC and older EC users, although tobacco flavour was used more in these groups than in younger users, people who used to smoke, or people with no history of combustible cigarette use. The authors hypothesise that the shift in the popularity to sweet flavours could be the result of a preference change or could reflect the increased availability of novel flavours on the market.

Whilst conducting the parent review, we have identified 64 ongoing intervention studies of EC for smoking cessation, potentially relevant for inclusion when complete.(1) None of these studies are RCTs designed to directly compare different EC flavours and measure our long-term outcomes. Therefore, further studies are needed to elucidate the relative effects of EC flavours. Currently, little evidence exists on the potential toxicity of different e-cigarette flavours, which we recognise is difficult to assess due to the large and evolving number of flavourings in use, and the multiple mediating and confounding factors, such as device type.(35) As more head-to-head trials comparing flavours become available, these should report on outcomes relevant to safety.

The limited and aggregated data in this review prevented a detailed examination of variables that may be mediating or confounding the relationship between flavour use and smoking cessation, for example, nicotine concentration, device power, e-liquid amount, and puff count. The investigation of these variables in future studies could improve understanding of the role flavours play in quitting smoking and find reasons for the heterogeneity between study effects identified in this review.

In conclusion, at the time of writing, intervention studies investigating EC for smoking cessation, with follow-up lengths of six months or longer, provide insufficient information on the popularity of EC flavours and their potential impacts on smoking cessation and long-term product use. Due to current uncertainties around the relative effects of flavours, future studies should aim to explore a broad range of flavours to inform ongoing policy debate and decisions around the regulation of EC flavours. Studies should also report data on important outcomes broken down by flavour type, and explore potential mediating and confounding factors, such as participant characteristics, nicotine concentration, device type, e-liquid use, and number of puffs. Long-term RCTs directly comparing the effects of different flavours on smoking and vaping behaviours, as well as exploring product safety are particularly needed. Based on the evidence that flavour experimentation takes place during studies, collecting detailed information about the flavours used by individual participants across the duration of studies is needed. Particular flavours may be favoured for achieving and others for maintaining abstinence. Although there is some limited evidence that sweet/fruit flavours may be more popular than tobacco or menthol/mint flavours, there appears to be deviations, which may reflect genuine individual or cultural differences across EC users.

**REFERENCES**

1. Lindson N, Butler AR, McRobbie H, Bullen C, Hajek P, Begh R, et al. Electronic cigarettes for smoking cessation. Cochrane Database of Systematic Reviews. 2024(1).

2. Glasser AM, Vojjala M, Cantrell J, Levy DT, Giovenco DP, Abrams D, et al. Patterns of e-cigarette use and subsequent cigarette smoking cessation over 2 years (2013/2014–2015/2016) in the population assessment of tobacco and health study. Nicotine and Tobacco Research. 2021;23(4):669-77.

3. Friedman AS, Xu S. Associations of flavored e-cigarette uptake with subsequent smoking initiation and cessation. JAMA network open. 2020;3(6):e203826-e.

4. Li L, Borland R, Cummings KM, Fong GT, Gravely S, Smith DM, et al. How Does the Use of Flavored Nicotine Vaping Products Relate to Progression Toward Quitting Smoking? Findings From the 2016 and 2018 ITC 4CV Surveys. Nicotine Tob Res. 2021;23(9):1490-7.

5. Mok Y, Jeon J, Levy DT, Meza R. Associations Between E-cigarette Use and E-cigarette Flavors With Cigarette Smoking Quit Attempts and Quit Success: Evidence From a U.S. Large, Nationally Representative 2018–2019 Survey. Nicotine & Tobacco Research. 2022;25(3):541-52.

6. Harlow AF, Fetterman JL, Ross CS, Robertson RM, Bhatnagar A, Benjamin EJ, et al. Association of device type, flavours and vaping behaviour with tobacco product transitions among adult electronic cigarette users in the USA. Tobacco Control. 2022;31(e1):e10-e7.

7. Gades MS, Alcheva A, Riegelman AL, Hatsukami DK. The role of nicotine and flavor in the abuse potential and appeal of electronic cigarettes for adult current and former cigarette and electronic cigarette users: A systematic review. Nicotine & Tobacco Research. 2022.

8. Gentry SV, Ward E, Dawkins L, Holland R, Notley C. Reported patterns of vaping to support long-term abstinence from smoking: a cross-sectional survey of a convenience sample of vapers. Harm reduction journal. 2020;17(1):1-9.

9. Liber AC, Knoll M, Cadham CJ, Issabakhsh M, Oh H, Cook S, et al. The role of flavored electronic nicotine delivery systems in smoking cessation: A systematic review. Drug and Alcohol Dependence Reports. 2023;7:100143.

10. Dyer ML, Khouja JN, Jackson AR, Havill MA, Dockrell MJ, Munafo MR, et al. Effects of electronic cigarette e-liquid flavouring on cigarette craving. Tobacco Control. 2023;32(e1):e3-e9.

11. Notley C, Gentry S, Cox S, Dockrell M, Havill M, Attwood AS, et al. Youth Use of E-Liquid Flavours–A systematic review exploring patterns of use of e liquid flavours and associations with continued vaping, tobacco smoking uptake, or cessation. Addiction. 2021.

12. Klein DE, Chaiton M, Kundu A, Schwartz R. A literature review on international e-cigarette regulatory policies. Current Addiction Reports. 2020;7(4):509-19.

13. Lindson N, Butler AR, Liber A, Levy DT, Barnett P, Theodoulou A, et al. An exploration of flavours in studies of e-cigarettes for smoking cessation: secondary analyses of a systematic review with meta-analyses. Addiction. 2023;118(4):634-45.

14. Hartmann-Boyce J, Lindson N. Assessing and minimizing risk of bias in randomized controlled trials of tobacco cessation interventions: Guidance from the Cochrane Tobacco Addiction Group. Addiction. 2023;118(9):1811-6.

15. Begh R, Coleman T, Yardley L, Barnes R, Naughton F, Gilbert H, et al. Examining the effectiveness of general practitioner and nurse promotion of electronic cigarettes versus standard care for smoking reduction and abstinence in hardcore smokers with smoking-related chronic disease: protocol for a randomised controlled trial. Trials. 2019;20(1):1-16.

16. Xu Y, Goldenson NI, Prakash S, Augustson EM, Shiffman S. Randomized trial assessing the effect of the JUUL system on switching away from cigarettes and smoking reduction among U.S. adults who smoke cigarettes. Exp Clin Psychopharmacol. 2024;32(1):3-15.

17. Dawkins L, Bauld L, Ford A, Robson D, Hajek P, Parrott S, et al. A cluster feasibility trial to explore the uptake and use of e-cigarettes versus usual care offered to smokers attending homeless centres in Great Britain. PloS one. 2020;15(10):e0240968.

18. Myers Smith K, Phillips‐Waller A, Pesola F, McRobbie H, Przulj D, Orzol M, et al. E‐cigarettes versus nicotine replacement treatment as harm reduction interventions for smokers who find quitting difficult: randomized controlled trial. Addiction. 2022;117(1):224-33.

19. Hajek P, Przulj D, Pesola F, Griffiths C, Walton R, McRobbie H, et al. Electronic cigarettes versus nicotine patches for smoking cessation in pregnancy: a randomized controlled trial. Nature Medicine. 2022;28(5):958-64.

20. Price AD, Coffey M, Houston L, Cook PA. Evaluation of a pharmacy supported e-cigarette smoking cessation intervention in Northwest England. BMC Public Health. 2022;22(1):1326.

21. Auer R, Schoeni A, Humair JP, Jacot-Sadowski I, Berlin I, Stuber MJ, et al. Electronic Nicotine-Delivery Systems for Smoking Cessation. N Engl J Med. 2024;390(7):601-10.

22. Caponnetto P, Campagna D, Maglia M, Benfatto F, Emma R, Caruso M, et al. Comparing the Effectiveness, Tolerability, and Acceptability of Heated Tobacco Products and Refillable Electronic Cigarettes for Cigarette Substitution (CEASEFIRE): Randomized Controlled Trial. JMIR Public Health Surveill. 2023;9:e42628.

23. Polosa R, Caponnetto P, Cibella F, Le-Houezec J. Quit and smoking reduction rates in vape shop consumers: a prospective 12-month survey. International journal of environmental research and public health. 2015;12(4):3428-38.

24. Holliday R, Preshaw PM, Ryan V, Sniehotta FF, McDonald S, Bauld L, et al. A feasibility study with embedded pilot randomised controlled trial and process evaluation of electronic cigarettes for smoking cessation in patients with periodontitis. Pilot and feasibility studies. 2019;5(1):1-14.

25. Pulvers K, Nollen NL, Rice M, Schmid CH, Qu K, Benowitz NL, et al. Effect of pod e-cigarettes vs cigarettes on carcinogen exposure among African American and Latinx smokers: a randomized clinical trial. JAMA network open. 2020;3(11):e2026324-e.

26. Goodwin RD, Ganz O, Weinberger AH, Smith PH, Wyka K, Delnevo CD. Menthol Cigarette Use Among Adults Who Smoke Cigarettes, 2008-2020: Rapid Growth and Widening Inequities in the United States. Nicotine Tob Res. 2023;25(4):692-8.

27. Bullen C, Howe C, Laugesen M, McRobbie H, Parag V, Williman J, et al. Electronic cigarettes for smoking cessation: a randomised controlled trial. The Lancet. 2013;382(9905):1629-37.

28. Hajek P, Phillips-Waller A, Przulj D, Pesola F, Myers Smith K, Bisal N, et al. A randomized trial of e-cigarettes versus nicotine-replacement therapy. New England Journal of Medicine. 2019;380(7):629-37.

29. Lee SM, Tenney R, Wallace AW, Arjomandi M. E-cigarettes versus nicotine patches for perioperative smoking cessation: a pilot randomized trial. PeerJ. 2018;6:e5609.

30. Russell C, McKeganey, Katsampouris E, Satchwell A, Haseen F. A randomised community-based trial of a closed-system pod e-vapour product and nicotine replacement therapy for cigarette abstinence and reduction [PH-353]. Society for Research on Nicotine and Tobacco (SRNT) 2021 Annual Meeting; February 24-27 2021; Virtual2021. p. 230.

31. Bansal-Travers M, Rivard C, Silveira ML, Kimmel H, Poonai K, Bernat JK, et al. Factors associated with changes in flavored tobacco products used: Findings from wave 2 and wave 3 (2014–2016) of the population assessment of tobacco and health (PATH) study. Addictive Behaviors. 2022;130:107290.

32. Zare S, Zheng Y. Consumer preferences for e-cigarette flavor, nicotine strength, and type: evidence from Nielsen scanner data. Nicotine and Tobacco Research. 2021;23(5):823-8.

33. Blank M-L, Hoek J. Choice and variety-seeking of e-liquids and flavor categories by New Zealand smokers using an electronic cigarette: A longitudinal study. Nicotine and Tobacco Research. 2021;23(5):798-806.

34. Jongenelis MI. E-cigarette product preferences of Australian adolescent and adult users: a 2022 study. BMC Public Health. 2023;23(1):220.

35. Barhdadi S, Rogiers V, Deconinck E, Vanhaecke T. Toxicity assessment of flavour chemicals used in e-cigarettes: current state and future challenges. Archives of Toxicology. 2021;95(8):2879-81.

36. Caponnetto P, Campagna D, Cibella F, Morjaria JB, Caruso M, Russo C, et al. EffiCiency and Safety of an eLectronic cigAreTte (ECLAT) as tobacco cigarettes substitute: a prospective 12-month randomized control design study. PloS one. 2013;8(6):e66317.

37. Carpenter MJ, Wahlquist AE, Dahne J, Gray KM, Cummings KM, Warren G, et al. Effect of unguided e-cigarette provision on uptake, use, and smoking cessation among adults who smoke in the USA: a naturalistic, randomised, controlled clinical trial. EClinicalMedicine. 2023;63:102142.

38. Cobb CO, Foulds J, Yen M-S, Veldheer S, Lopez AA, Yingst JM, et al. Effect of an electronic nicotine delivery system with 0, 8, or 36 mg/mL liquid nicotine versus a cigarette substitute on tobacco-related toxicant exposure: a four-arm, parallel-group, randomised, controlled trial. The Lancet Respiratory Medicine. 2021;9(8):840-50.

39. Eisenberg MJ, Hébert-Losier A, Windle SB, Greenspoon T, Brandys T, Fülöp T, et al. Effect of e-cigarettes plus counseling vs counseling alone on smoking cessation: a randomized clinical trial. JAMA. 2020;324(18):1844-54.

40. Ely J. Evaluation of the use of electric cigarettes in a rural smoking cessation program: University of Northern Colorado. Available at: <https://digscholarship.unco.edu/cgi/viewcontent.cgi?article=1001&context=capstones> [Accessed 10 May 2022]; 2013.

41. Halpern SD, Harhay MO, Saulsgiver K, Brophy C, Troxel AB, Volpp KG. A pragmatic trial of e-cigarettes, incentives, and drugs for smoking cessation. New England Journal of Medicine. 2018;378(24):2302-10.

42. Klonizakis M, Gumber A, McIntosh E, Brose LS. Medium- and longer-term cardiovascular effects of e-cigarettes in adults making a stop-smoking attempt: a randomized controlled trial. BMC Medicine. 2022;20(1):276.

43. Lucchiari C, Masiero M, Mazzocco K, Veronesi G, Maisonneuve P, Jemos C, et al. Benefits of e-cigarettes in smoking reduction and in pulmonary health among chronic smokers undergoing a lung cancer screening program at 6 months. Addictive behaviors. 2020;103:106222.

44. Pope I, Clark LV, Clark A, Ward E, Belderson P, Stirling S, et al. Cessation of Smoking Trial in the Emergency Department (COSTED): a multicentre randomised controlled trial. Emergency Medicine Journal. 2024;41(5):276-82.

45. Pratt SI, Ferron JC, Brunette MF, Santos M, Sargent J, Xie H. E-Cigarette Provision to Promote Switching in Cigarette Smokers With Serious Mental Illness-A Randomized Trial. Nicotine Tob Res. 2022;24(9):1405-12.

*Table 1: Characteristics of included studies*

| **Study ID** | **Device type** | **Total N baseline** | **Flavours provided** | **Comparison (C) or single arm (S)** | **Study design** | **Length of follow-up (months)** | **Overall risk of bias judgement** | **Country** | **Population characteristics** |
| --- | --- | --- | --- | --- | --- | --- | --- | --- | --- |
| Auer 2024*(21) | Refillable | 1246 | Choice of sweet, tobacco or menthol | C (EC vs counselling) | RCT | 6 | High | Switzerland | People who smoke combustible cigarettes |
| Begh 2021(15) | Refillable | 325 | Choice of sweet, tobacco or menthol | C (EC vs standard care) | RCT | 8 | High | UK | People who smoke combustible cigarettes with no plans to stop |
| Bullen 2013(27) | Cig-a-like | 657 | Tobacco only | C (EC vs nicotine patches vs placebo EC) | RCT | 6 | Low | New Zealand | People who smoke combustible cigarettes and willing to quit |
| Caponnetto 2013(36) | Cig-a-like | 300 | Tobacco only | C (EC vs lower nicotine EC vs non-nicotine EC) | RCT | 12 | Unclear | Italy | People who smoke combustible cigarettes |
| Caponnetto 2023*(22) | Refillable | 220 | Choice of tobacco or menthol | C (EC vs heated tobacco) | RCT | 6 | High | Italy | People who smoke combustible cigarettes |
| Carpenter 2023*(37) | Pod | 638 | Choice of sweet, tobacco or menthol | C (EC vs no intervention) | RCT | 6 | High | USA | People who smoke combustible cigarettes |
| Cobb 2021(38) | Cartridge | 520 | Choice of tobacco or menthol | C (EC nicotine 2 strengths; non-nicotine EC; cigarette substitute) | RCT | 6 | Low | USA | People who smoke combustible cigarettes |
| Dawkins 2020(17) | Refillable | 80 | Choice of sweet, tobacco or menthol | C (EC vs UC) | Prospective cohort | 6 | High | UK | People who smoke combustible cigarettes. Recruitment at homeless centres |
| Eisenberg 2020(39) | Cig-a-like | 376 | Tobacco only | C (EC + counselling vs non-nicotine EC + counselling vs counselling only) | RCT | 6 | Low | Canada | People who smoke combustible cigarettes and motivated to quit |
| Ely 2013(40) | Cig-a-like | 48 | Choice of sweet, tobacco or menthol | S (All used EC) | Prospective cohort | 6 | High | USA | People who want to quit combustible cigarettes or switch to EC |
| Hajek 2019(28) | Refillable | 886 | Tobacco only | C (EC vs NRT) | RCT | 12 | Low | UK | People who smoke combustible cigarettes |
| Hajek 2022*(19) | Refillable | 1140 | Choice of sweet or tobacco | C (EC vs NRT) | RCT | 6 | Low | UK | People who smoke combustible cigarettes, 12-24 weeks pregnant |
| Halpern 2018(41) | Cig-a-like | 6006 | Choice of sweet, tobacco or menthol | C (Usual care (UC); UC +EC; UC+EC+ NRT + bupropion or varenicline; UC+EC+ NRT + bupropion or varenicline + incentives; as before plus financial incentive) | RCT | 12 | High | USA | People who smoke and employees and their spouses that used Vitality wellness programs |
| Holliday 2019(24) | Refillable | 80 | Choice of sweet, tobacco, mint/menthol, or unflavoured | C (EC vs no intervention) | RCT | 6 | High | UK | People who smoke combustible cigarettes with peridontis |
| Klonizakis 2022*(42) | Cartridge | 248 | Choice of tobacco or menthol | C (EC vs non-nicotine EC; EC vs NRT) | RCT | 6 | Unclear | UK | People who smoke combustible cigarettes |
| Lee 2018(29) | Cig-a-like | 30 | Tobacco only | C (EC vs nicotine patches) | RCT | 6 | Low | USA | People who smoke and presented to the anesthesia preoperative clinic for elective surgery 3 or more days before surgery |
| Lucchiari 2020(43) | Cig-a-like | 210 | Tobacco only | C (nicotine EC vs non-nicotine EC) | RCT | 12 but data only available at 6 | High | Italy | Participants are 55 years or more and have smoked at least 10 combustible cigarettes a day for the past 10 years |
| Myers Smith 2022(18) | Refillable | 135 | Choice of sweet, tobacco or menthol | C (EC vs NRT) | RCT | 6 | Low | UK | People who smoke combustible cigarettes and find quitting difficult |
| Polosa 2015(23) | Refillable | 71 | Choice of sweet, tobacco or menthol | S (All used EC) | Prospective cohort | 12 | High | Italy | People who smoke combustible cigarettes, making first purchase at vape shop |
| Pope 2024*(44) | Pod | 972 | Choice of sweet, tobacco or menthol | C (EC vs UC) | RCT | 6 | High | UK | People attending the Emergency Department who smoked tobacco daily |
| Pratt 2022*(45) | Cartridge | 240 | Choice of tobacco or menthol | C (EC vs no intervention) | RCT | 6 | High | USA | People who smoke combustible cigarettes with serious mental illness |
| Price 2022*(20) | Refillable | 871 | Choice of sweet, tobacco or menthol | S (All offered EC) | Single-arm intervention study | 12 | High | UK | People who smoke combustible cigarettes from lower socioeconomic groups |
| Pulvers 2020(25) | Pod | 186 | Choice of sweet, tobacco or menthol | C (EC versus no intervention) | RCT | 6 | High | USA | African American and Latinx people who smoke combustible cigarettes |
| Russell 2021(30) | Pod | 426 | Choice of sweet, tobacco or menthol | C (NRT; EC with nicotine salt e‐liquid pods; EC with freebase nicotine e‐liquid pods) | RCT | 6 | Unclear | UK | People who smoke combustible cigarettes |
| Xu 2023*(16) | Pod | 837 | 1. Tobacco only; 2. Choice of sweet, tobacco or menthol | C (Tobacco flavour EC vs choice of flavour EC vs quit advice) | RCT | 12 | High | USA | People who smoke combustible cigarettes |

Footnote: EC = electronic cigarettes; NRT = nicotine replacement therapy; RCT = randomised controlled trial; UC = usual care; *new to this update

*Table 2. Flavours available and participant choices in studies that offered a range of flavours*

| **Study ID** | **Country** | **Flavour choices available (verbatim where possible)** | **Flavour category** | **Choice data** |
| --- | --- | --- | --- | --- |
| Auer 2024(21) | Switzerland | "We offered ENDS and six different e-liquid flavors (two tobacco flavors, menthol, green apple, raspberry, and red fruits)" | Choice of sweet, tobacco or menthol | “For intervention group (N= 620). At week 1, the proportions of participants reporting use of tobacco flavored e-liquids were similar between ENDS only and dual users (24% of 409 ENDS only vs. 27% of 129 dual users, p=.58). Therefore, 98/409 ENDS only and 35/129 dual users, meaning 133 of the 538 (25%) who gave info on e-liquid reported using tobacco flavour at 1 week. At month 6, ENDS only users reported fewer tobacco flavors than dual users (21% of 266 ENDS only vs. 33% of 102 dual users, RR 0.49, p<.001). Therefore, 56/266 ENDS only and 34/102 dual users, meaning 90 of the 368 (25%) who gave info on e-liquid reported using tobacco flavour at 6 months.” "Other flavor choices among the two groups were similar." |
| Begh 2021(15) | UK | “The starter pack will contain three different flavoured bottles of e-liquid […]. Participants will need to purchase their own e-liquid thereafter” | Choice of sweet, tobacco or menthol | At 2 months (N = 111): blueberry n = 15 (13.5%); forest fruit n = 28 (25.25%); strawberry n = 5 (4.5%), other fruit flavours n = 20 (18%); menthol n = 10 (9%), tobacco n = 21 (18.9%), unflavoured n = 1 (0.9%), other flavours (blackjack, bubblegum, CBD oil, coffee, ginger, liquorice, mint, nicotine, toffee, vanilla) n = 11 (9.9%)  At 8 months (N = 32): blueberry n = 6, (18.8%); forest fruit n = 4 (12.5%), other fruit flavour n = 1 (3.1%), menthol n = 11 (34.4%), tobacco n = 10 (31.3%) |
| Caponnetto 2023(22) | Italy | "Participants could choose 1 out of 3 different flavors [sweet tobacco, full tobacco or menthol...] and were provided with their preferred flavor for the whole duration of the study." | Choice of tobacco or menthol | 50.9% (56/110) chose Puff Riserva Country (sweet tobacco), 30.9% (34/110) chose Puff Riserva Tuscan (full tobacco), and 18.2% (20/110) chose Puff Artic (menthol) e-liquid |
| Carpenter 2023(37) | USA | "Participants could choose up to 2 flavors among 5 offered: tobacco, menthol, blue/ blackberry (one flavor), apple melon, or iced fruit, and could change selection at 2nd shipment." | Choice of sweet, tobacco or menthol | Not reported |
| Cobb 2021(38) | USA | “participant-selected at randomisation: tobacco or menthol; selected flavour could not be changed during the intervention period” | Choice of tobacco or menthol | Not reported |
| Dawkins 2020(17) | UK | “3 options: tobacco, fruit, menthol […] Participants were given time to try different flavours and nicotine strengths at baseline and be permitted to switch between flavours in accordance with documented vaping practices” | Choice of sweet, tobacco or menthol | Across the duration of the study, 318 bottles of fruit flavoured 10 ml e-liquid were dispensed; 155 bottles of menthol and 133 bottles of tobacco. |
| Ely 2013(40) | USA | “If the participant chooses to try e-cigarettes […] Information on the two chosen e-cigarettes (BluCig and SmokeTip) that were used in this program regarding cost, nicotine doses and flavors, […] was provided” | Choice of sweet, tobacco or menthol | Not reported |
| Hajek 2022(19) | UK | "Participants were sent […] two 10 ml bottles of tobacco-flavored e-cigarette liquid [...]. Further supplies of e-cigarette liquid were posted on request for up to 8 weeks. A lower strength e-cigarette liquid (1.1%) and e-cigarette liquid with fruit flavor were available as alternatives. Participants were encouraged to source e-cigarette liquids of the strength and flavor they liked, as well as different e-cigarette devices, and arrange their own supplies after 8 weeks if needed." "Most used e-cigarette liquids [...] with tobacco and fruit flavors." | Choice of sweet or tobacco | Products used during the initial 4 weeks (N = 344) - Fruit: 180 (52.3); Tobacco: 24 (7.0); Mint/menthol: 22 (6.4); Chocolate, dessert, candy: 11 (3.2); Other: 21 (6.1); Information missing: 86 (25.0). Products used since last contact at End of Pregnancy (N = 371)- Fruit: 97 (26.2); Mint/menthol: 38 (10.2); Chocolate, dessert, candy: 19 (5.1); Tobacco: 17 (4.6); Other: 24 (6.5); Information missing: 176 (47.4) |
| Halpern 2018(41) | USA | “e-cigarettes […] in participants’ chosen flavors” | Choice of sweet, tobacco or menthol | Not reported |
| Holliday 2019(24) | UK | “participants were provided with an approximately 2-week supply of e-liquid (with a choice of flavour [tobacco, mint, cherry or unflavoured] and nicotine strength) and information on where to buy more.” | Choice of sweet, tobacco, mint/menthol, or unflavoured | Total N = 39. Cherry only n = 4 (10%); mint only n = 8 (21%); mint and cherry n = 6 (15%); tobacco only n = 5 (13%); tobacco and cherry n = 2 (5%); tobacco and mint n = 9 (23%); unflavoured only n = 0; unflavoured and tobacco n = 3 (8%); unflavoured and mint n = 1 (3%); unflavoured and cherry n = 1 (3%) |
| Klonizakis 2022(42) | UK | "Participants could choose ice menthol or tobacco flavor (Red Label, Totally Wicked, Blackburn, UK)." | Choice of tobacco or menthol | Not reported |
| Myers-Smith 2022(18) | UK | Participants “were instructed to obtain [an e-cigarette] of their choice, together with initial samples of e-liquid with the strength and flavour of their choice” | Choice of sweet, tobacco or menthol | At 1 week (N = 49): fruit: n = 21; sweet: 5; energy/soft drink: 2; coffee: 3; menthol/mint: 8; tobacco: 13; unknown other: 6 (multiple flavours used by some)  At 6 months (N = 31): fruit: 18; sweet: 2; energy/soft drink: 2; menthol/mint: 5; tobacco: 6; raspberry and mint: 1; coffee and coconut: 1 |
| Polosa 2015(23) | Italy | “Participating shops […] displayed a […] large selection of flavours’ | Choice of sweet, tobacco or menthol | Baseline (N = 71): fruit n = 4 (5.6%); mint n = 7 (9.9%); tobacco n = 57 (80.3%); unknown other n = 3(4.2%)  At 12 months (N = 49): fruit n = 2 (4.1%); mint n = 5 (10.2%); tobacco n = 36 (73.5%); other flavours (cola, coffee, dessert/cakes/cookies, cocktail, mixed berry, mint) n = 6 (12.3%). |
| Pope 2024(44) | UK | "The kit included 11 pods (3 tobacco flavoured, 4 berry flavoured and 4 menthol flavoured) of 20mg/mL nicotine strength." | Choice of sweet, tobacco or menthol | Not reported |
| Pratt 2022(45) | USA | “We offered two tobacco flavours and menthol” (from personal communication) | Choice of tobacco or menthol | Not reported |
| Price 2022(20) | UK | "The most popular flavour was mixed fruit, accounting for nearly half (49%) of the bottles distributed, followed by menthol (23%), tobacco (17%) and rolled tobacco (11% of bottles)" | Choice of sweet, tobacco or menthol | "During the pilot programme, a total of 3442 bottles of liquid were distributed, an average of just over 4 bottles per person taking part. The most popular flavour was mixed fruit, accounting for nearly half (49%) of the bottles distributed, followed by menthol (23%), tobacco (17%) and rolled tobacco (11% of bottles)." |
| Pulvers 2020(25) | USA | “Those randomized to the e-cigarette group received a JUUL e-cigarette and pods in a choice of flavor” [menthol, mint, mango, or tobacco] | Choice of sweet, tobacco or menthol | Baseline (N = 125): mango n = 35 (28%); menthol n = 44 (35.2%); mint n = 24 (19.2%); tobacco n = 22 (17.6%)  At 2 and 6 weeks (N = 113): mango n = 36 (31.9%); menthol n = 39 (34.5%); mint n = 17 (15%); tobacco n = 21 (18.6%). |
| Russell 2021(30) | UK | “Participants […] were given a […] device, and reimbursement for retail purchases of up to 12 e-liquid pods (six packs of x2 pods) per month for three months. Participants were encouraged […] to choose and change flavours […] as they wished.” | Choice of sweet, tobacco or menthol | Not reported |
| Xu 2023(16) | USA | "participants were randomized to receive either: (a) JUUL with only Virginia Tobacco flavor; (b) JUUL with a choice of flavors or (c) quit advice" | Tobacco and choice of sweet, tobacco or menthol | N with any use of flavour in past 30 days at 12 month follow up:  Virginia tobacco arm: Virginia tobacco: 56, classic tobacco: 24, menthol: 22, mint: 19, mango: 13, crème: 7, fruit: 15, cucumber: 2.  Flavour choice arm: Virginia tobacco: 37, classic tobacco: 26, menthol: 20, mint: 37, mango: 40, crème: 28, fruit: 18, cucumber: 3. |

*Table 3. Results from random-effects meta-analyses subgrouped by flavour*

| **Comparison** | **Outcome** | **Number of studies** | **I^2^ for subgroup differences (%)** | **P value for subgroup differences** |
| --- | --- | --- | --- | --- |
| Nicotine EC versus NRT | Smoking cessation | 3 tobacco only; 1 choice of tobacco or menthol; 1 choice of tobacco or sweet; 2 choice of tobacco, menthol or sweet | 0 | 0.91 |
|  | Study product use (Figure 2) | 3 tobacco only; 2 choice of tobacco, menthol or sweet | 65.2 | 0.09 |
| Nicotine EC versus non-nicotine EC | Smoking cessation | 4 tobacco only; 2 choice of tobacco or menthol | 0 | 0.68 |
|  | Study product use | 2 tobacco only; 1 choice of tobacco or menthol | 0 | 0.86 |
| Nicotine EC versus behavioural support only or no support | Smoking cessation | 3 tobacco only; 1 choice of tobacco or menthol; 8 choice of tobacco, menthol or sweet | 0 | 0.63 |

Footnote: EC = electronic cigarettes; NRT = nicotine replacement therapy

*Figure 1. Flavour choice over time in five studies providing options including tobacco, mint/menthol and sweet flavours*


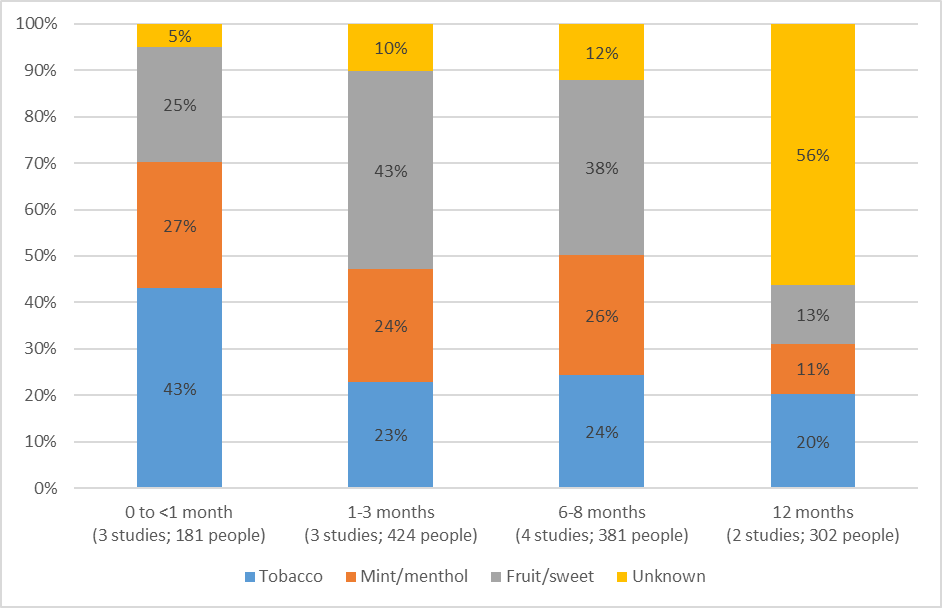


Figure 2. EC flavour use over time among participants in a) Pulvers 2020,(25) b) Xu 2023.(16) Arrows illustrate the flow of flavour choice and switching behaviour (only including participants that provided data at follow-up points)


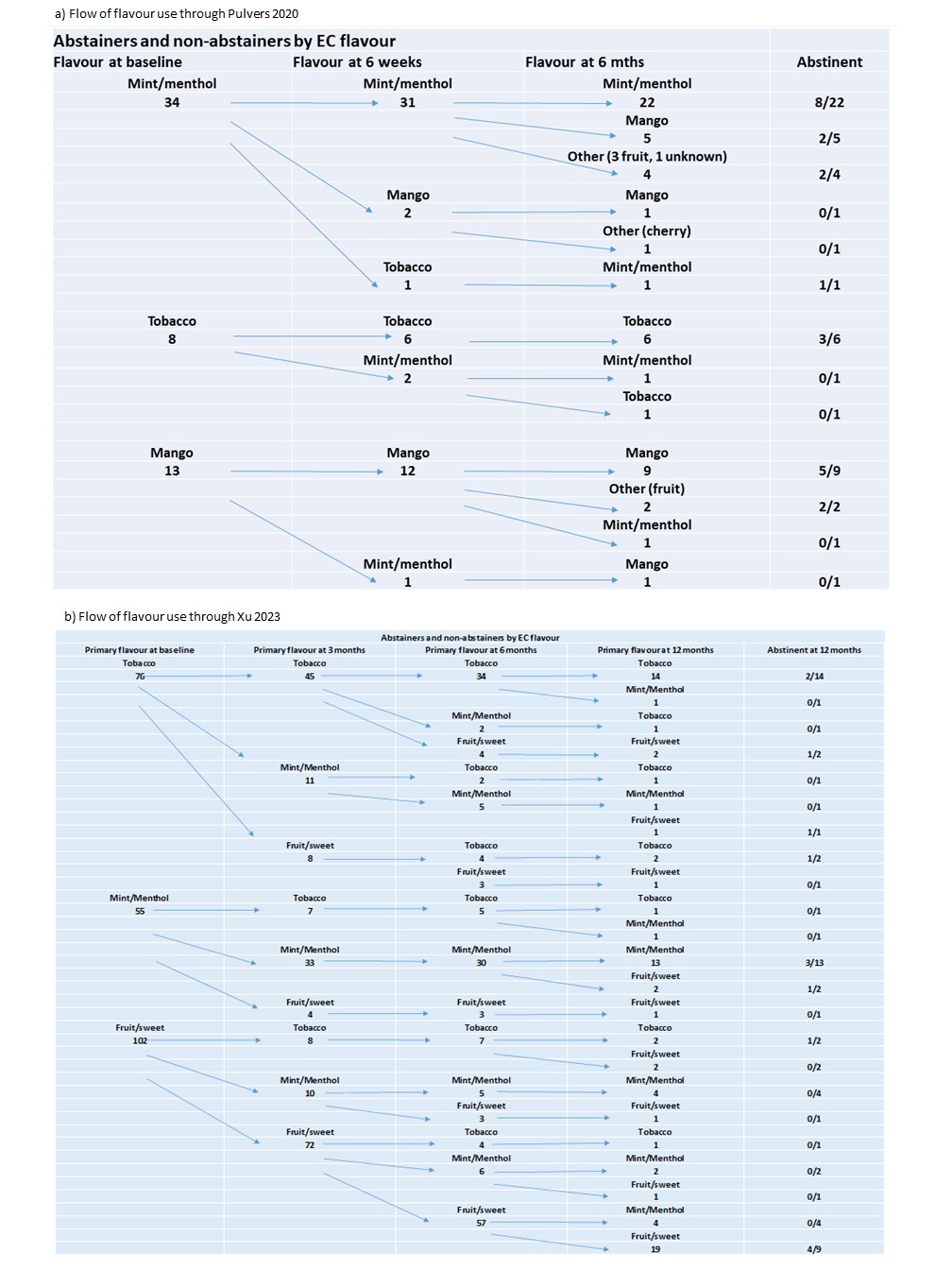


Footnote: a) at baseline and 6-week follow-up participants were provided with mango, mint, menthol or tobacco flavours. At 6-month follow-up participants were self-sourcing flavours and so additional flavours were being used, as specified; b) up to 6 months participants were supplied with tobacco, mint, menthol, mango, creme, fruit or cucumber by the study; at 12 months participants were self-sourcing EC supplies

*Figure 3. Study product use at 6 months or longer, EC versus NRT*


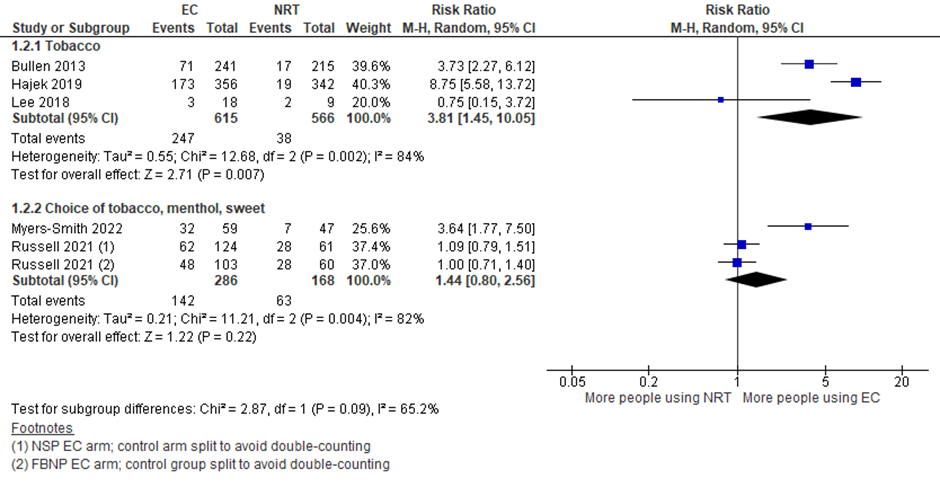


Footnote: EC = electronic cigarettes; FBNP = free base nicotine pods; NSP = nicotine salt pods; NRT = nicotine replacement therapy

*Figure 4. EC flavour use among a) people abstinent from combustible cigarettes at longest follow-up b) people continuing to use combustible cigarettes at longest follow-up*
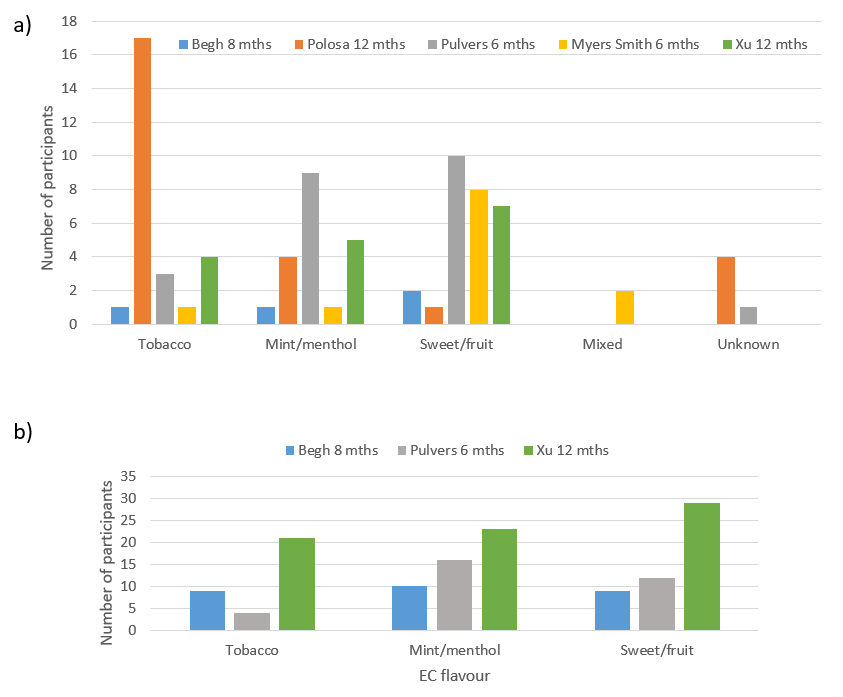


Footnote: in the ‘mixed’ flavour category, one participant was using both coconut and coffee flavoured e-liquids and one participant was using both raspberry and mint flavours
